# Broken chromosomes heading into mitosis: More than one way to patch a flat tire

**DOI:** 10.1083/jcb.202401085

**Published:** 2024-03-13

**Authors:** C. Luke Messer, Donald T. Fox

**Affiliations:** 1 Department of Biology, St. Bonaventure University, St. Bonaventure, NY, USA; 2 Department of Pharmacology and Cancer Biology, Duke University Medical Center, Durham, NC, USA; 3 Duke Regeneration Center, Duke University Medical Center, Durham, NC, USA; 4 Duke Cancer Institute, Duke University Medical Center, Durham, NC, USA

## Abstract

A cell dealing with a broken chromosome in mitosis is like a driver dealing with a flat tire on the highway: damage repair must occur under non-ideal circumstances. Mitotic chromosome breaks encounter problems related to structures called micronuclei. These aberrant nuclei are linked to cell death, mutagenesis, and cancer. In the last few years, a flurry of studies illuminated two mechanisms that prevent mitotic problems related to micronuclei. One mechanism prevents micronuclei from forming during mitosis and involves DNA Polymerase Theta, a DNA repair regulator that patches up broken mitotic chromosomes. A second mechanism is activated after micronuclei form and then rupture, and involves CIP2A and TOPBP1 proteins, which patch micronuclear fragments to promote their subsequent mitotic segregation. Here, we review recent progress in this field of mitotic DNA damage and discuss why multiple mechanisms exist. Future studies in this exciting area will reveal new DNA break responses and inform therapeutic strategies.

## Teaser

Messer and Fox discuss new work that highlights the multiple molecular mechanisms by which cells respond to mitosis-related DNA damage and the danger presented by the micronucleus.

## Mitosis: DNA damage on a road trip

Double-stranded DNA breaks (DSBs) challenge genome stability. DSBs often occur within an interphase nucleus, and these aberrations activate a host of DNA damage checkpoint responses (DDRs). Such canonical DDRs ultimately activate either apoptosis or cell cycle arrest and DSB repair. Interphase nuclear DSB repair primarily occurs by one of two canonical pathways: homologous recombination (HR) or non-homologous end joining (NHEJ) (Waterman et al., 2020).

Think of this repair as discovering a gash in your car tire while the car is in your driveway—you halt all plans to drive the car and deal with the gash rather than risk future catastrophe. In the same way, interphase nuclear DSB repair gives the cell time to properly correct a potentially catastrophic genomic insult.

Now imagine that the gash instead occurs while you are driving. This is not an ideal time and place for a thorough repair. Your best options may involve a temporary patch of the damage, just enough to get you to a better repair location. Mitosis is analogous to such a situation: chromosomes are highly condensed and rapidly segregating toward spindle poles, and interphase DSB responders may be unavailable (Giunta et al., 2010; Orthwein et al., 2014; van Vugt et al., 2010). The idea that interphase DDRs are unavailable in mitosis stems from how such responses are mechanistically wired as interphase DNA damage halts the cell cycle before preceding to the next phase (such as G2 arrest before M phase) (Yam et al., 2022; Clay and Fox, 2021).

Luckily for the mitotic cell, all is not hopeless when DSBs associate with cell division. In at least some contexts, mechanisms exist to halt or delay mitotic progression in response to DSBs (Mikhailov et al., 2002; Royou et al., 2005; Yang et al., 1997; Kim and Burke, 2008). Several recent studies have added to our understanding of mitosis-associated DDRs in important ways. While this new work highlights multiple molecular mechanisms to respond to mitosis-related DNA damage, a commonality is the danger presented by a structure known as a micronucleus.

**Figure 1.**
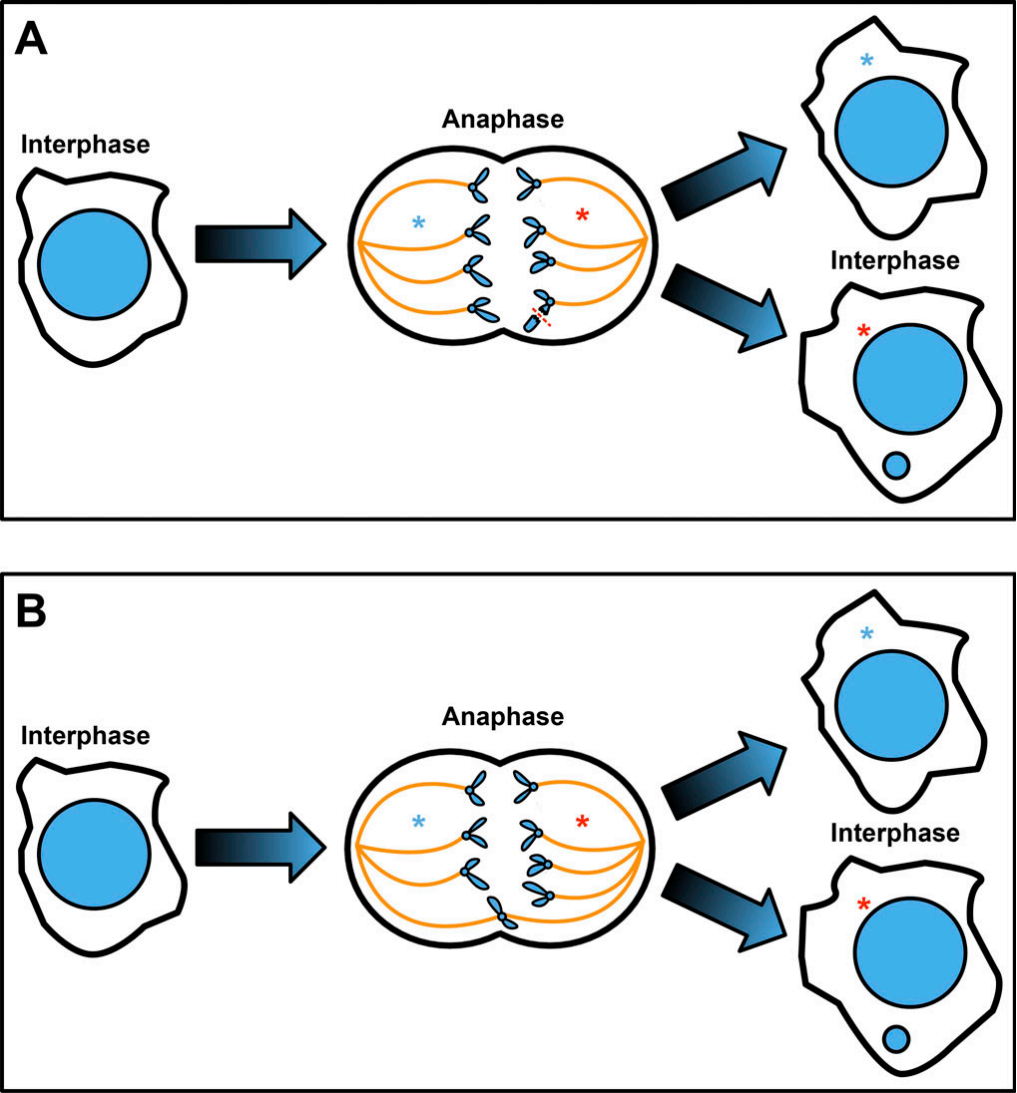
**Micronucleus formation during mitosis.** Panels depict one round of cell division and the preceding and subsequent interphase. DNA, blue (large circle = nucleus, small circle = micronucleus, and smaller V-shaped structures = chromatids), spindle microtubules, yellow. Blue and red asterisks track each presumptive and resulting daughter cell. **(A)** DNA breakage and resulting micronucleus. **(B)** Anaphase with aberrant spindle attachments and resulting micronucleus.

The micronucleus is a cytosolic DNA that forms during mitosis when part of the genome (one or more chromosomes or chromosome fragments) fails to incorporate into newly formed daughter nuclei (Fig. 1). Micronuclei cause cell death (Swanson et al., 1995; Utani et al., 2010), aneuploidy (Vázquez-Diez et al., 2016; He et al., 2019), and a genome-shattering event known as chromothripsis (Kato and Sandberg, 1968; Zhang et al., 2015; Crasta et al., 2012; Hatch and Hetzer, 2015). Micronuclei also upregulate cyclic GMP-AMP synthase (cGAS), an immune regulator linked to tumorigenesis (MacKenzie et al., 2017; Liu et al., 2018; Bakhoum et al., 2018). Micronuclei are thought to have defective nucleo-cytoplasmic transport as these structures replicate out of synch with the rest of a cell’s genome, exhibit defects in DNA repair, and exhibit compromised membrane integrity (Crasta et al., 2012; Zhang et al., 2015; Hatch and Hetzer, 2015).

It is in each cell’s best interest to prevent micronuclei from forming and/or rupturing, and mitosis appears to be especially vulnerable. Here, we focus on recent studies that reveal two fail-safe mechanisms that prevent micronuclei from wreaking havoc in a mitotic cell. The first mechanism prevents micronuclei from forming during mitosis in the first place. The second mechanism acts after a micronucleus has already formed and quickly acts upon interphase micronucleus rupture to patch up broken DNA into a single structure. This structure then facilitates its subsequent mitotic segregation and reencapsulation in the nucleus, effectively eliminating the micronucleus. These studies expand our understanding of DDRs in the context of mitosis and are thus important to studying cell proliferation in organ development, regeneration, and cancer.

### Patch #1: Microhomology-mediated end joining

Intact mitotic chromosomes that are correctly attached to the mitotic spindle will rapidly segregate to spindle poles during anaphase. In contrast, broken chromosomes (Fig. 1 A) or aberrant spindle attachments (Fig. 1 B) can often lead to lagging DNA that fails to segregate or segregates more slowly. Such lagging DNA is at risk of forming micronuclei (Fig. 1).

Cells can act during mitosis to prevent new micronuclei. Recent studies have highlighted a role in this process for DNA repair by microhomology-mediated end joining (MMEJ). MMEJ is also frequently referred to as either alternative end joining or Theta-mediated end joining, reflecting the central role of DNA Polymerase Theta (Pol Theta) in this process. These new studies show that MMEJ responds to DNA breaks that persist into mitosis, thereby preventing new micronuclei.

MMEJ involves DNA annealing at areas of microhomology. This repair process generates signature 10–60 base pair (bp) deletions (Ramsden et al., 2022). MMEJ is frequently referred to as a “backup” DNA repair process. This is because cells with interphase nuclear DNA damage typically utilize either HR or NHEJ before cell division. The MMEJ regulator POL THETA is frequently synthetic lethal in cells that are deficient for DNA repair by HR or NHEJ (Brambati et al., 2023; Feng et al., 2019; Fleury et al., 2023), indicating a heightened role for MMEJ in cells deficient for interphase DDRs. Further, many cancer cells upregulate POL THETA expression (Barszczewska-Pietraszek et al., 2022), which may indicate that cancers rewire DDRs to be more reliant on MMEJ.

Roles for MMEJ in the context of DSBs that persist into mitosis have been revealed from several recent studies that focus on cells lacking interphase DDR regulators. In human HeLa cells depleted of the HR factors BRCA2 or RAD51, POL THETA activity was required in mitosis to respond to DNA breaks that were not repaired in the preceding interphase. These experiments and others collectively indicated that HR factors can suppress MMEJ until the onset of mitosis (Llorens-Agost et al., 2021a, 2021b).

**Figure 2.**
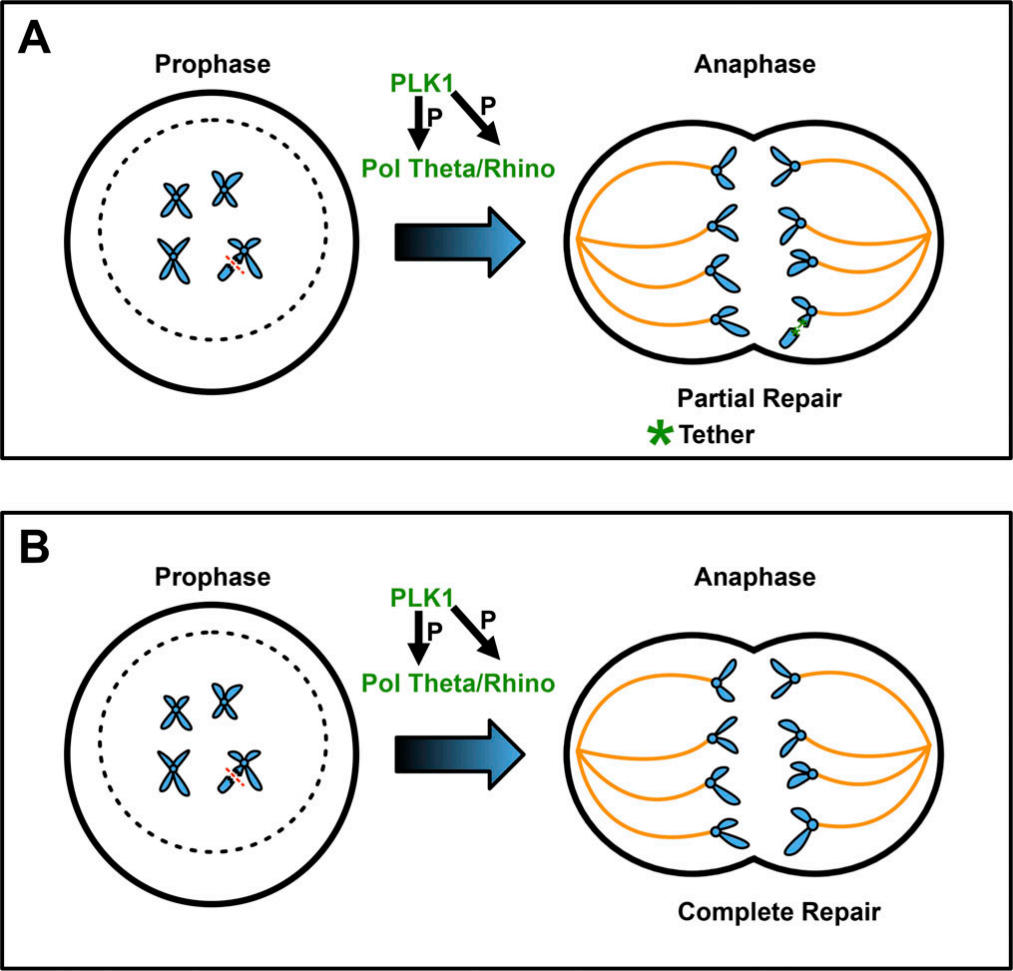
**Mitotic MMEJ. (A and B)** Panels depict a mitotic DSB (red dashed line) that is either partially (A) or fully (B) repaired during mitosis by PLK1 phosphorylation of POL THETA and RHINO, which promotes POL THETA/RHINO interaction. DNA, blue; spindle microtubules, yellow; repair tether, green; dashed circle, nuclear envelope breaking down.

Similarly, in *Drosophila* intestinal papillar cells that do not activate interphase cell cycle arrest or apoptosis in response to DSBs, Pol Theta was found to be critical in responding to DSBs that persist into mitosis (Bretscher and Fox, 2016; Clay et al., 2021). This activity of Pol Theta promotes poleward segregation of DNA fragments without centromeres (acentric fragments), likely by annealing the broken fragment to centromere containing DNA (Fig. 2 A). Evidence for this annealing model comes from examining Mre11, an early responder to DNA breakage that resects DNA to expose single strands at either end of a DSB, and Rpa3, which binds resected single-stranded DNA at DSBs. As papillar cells with DSBs enter mitosis, Mre11 and then Rpa3 are removed from the DSB in a stepwise manner as the acentric DNA segregates. As Pol Theta-catalyzed MMEJ is known to displace Rpa from sites of DNA breakage once resected single DNA strands from opposite sides of a DSB anneal (Mateos-Gomez et al., 2017), these results suggested that MMEJ annealing between the centromeric and acentric side of the DSB is sufficient to promote poleward segregation of acentric DNA. In papillar cells lacking Pol Theta, Rpa3 instead persists on acentric DNA, which then forms a micronucleus. Many cells with micronuclei then die, which causes intestinal organ malformation (Clay et al., 2021). These studies underscore an important role for MMEJ in mitosis, especially in the context of cells that are deficient for interphase DDRs.

While the above studies link Pol Theta to mitotic DSB responses, MMEJ is also active in interphase. This begs the question of how MMEJ can be specifically activated in mitosis. Two very recent studies have shed light on molecular mechanisms, whereby Pol Theta is recruited to mitotic DSBs. In cultured human cells, Gelot et al. (2023) identified a critical role for the mitotic kinase Polo-like kinase 1 (PLK1) in phosphorylating POL THETA (Gelot et al., 2023), while Brambati et al. (2023) identified the RHINO protein as a critical regulator of mitotic MMEJ (Brambati et al., 2023).

Gelot et al. (2023) examined the cell cycle distribution of POL THETA repair foci in control versus HR-deficient human cells (several lines including retinal pigment and embryonic kidney) with DSBs (Gelot et al., 2023). Whereas control cells contained mostly S-phase POL THETA repair foci, foci in HR-deficient cells spanned the period from G2 through M-phase and into the next G1. Immunoprecipitation of POL THETA in HR-deficient cells ultimately revealed mitosis-specific POL THETA phosphorylation, and this phosphorylation was abolished by two different PLK1 inhibitors. Using a Cas9 DSB system, the authors then showed that mitotic DSBs are repaired by POL THETA. Sequencing of repair products revealed that the absence of POL THETA abolished the 10–60 base pair size deletions at DSB sites, which are a signature of MMEJ. In cells expressing a form of POL THETA that cannot be phosphorylated by PLK1, HR-deficient cells undergo high rates of mitotic cell death or form micronuclei, similar to findings in *Drosophila* papillar cells lacking Pol Theta (Clay et al., 2021).

Notably, Gelot et al. (2023) found that many POL THETA foci persisted into the following G1 in nuclear bodies, which the authors suggest may represent continued repair following mitosis (again consistent with findings in *Drosophila*) (Gelot et al., 2023). This observation is consistent with the idea that MMEJ may in some instances function as a “repair patch” that enables cells to survive mitosis prior to more complete repair of any remaining DNA damage that may occur in the following G1 phase (Fig. 2 A). This study highlights the role of mitotic phosphorylation by PLK1 of the MMEJ regulator POL THETA to catalyze at least partial MMEJ annealing to prevent micronuclei.

Brambati et al. (2023) used a synthetic lethality screen for cell survival factors in HR and NHEJ-deficient human cells, which lacked the repair factors BRCA2, LIG4, and TP53 (Brambati et al., 2023). POL THETA was found in this screen, in agreement with previous studies. Additionally, members of the DNA repair complex known as 9-1-1 (encoded by RAD9A, RAD1, and HUS1) and a known interacting partner, RHINO (Cotta-Ramusino et al., 2011), were also identified as required in the absence of HR and NHEJ. Coimmunoprecipitation experiments in embryonic kidney cells provided direct evidence of an interaction between RHINO protein and POL THETA, specifically during mitosis, when RHINO levels and phosphorylation accumulate. Moreover, live imaging experiments using Halo-tagged POL THETA indicated a function for RHINO in POL THETA recruitment. Importantly, DSB induction through irradiation increased micronuclei after mitosis in cells lacking POL THETA and RHINO.

As Gelot et al. (2023) found a role for PLK1 phosphorylation of POL THETA to activate mitotic MMEJ, Brambati et al. (2023) showed that PLK1 additionally phosphorylates RHINO in this context (Brambati et al., 2023). Specifically, PLK1 inhibition reduced RHINO phosphorylation, and changing several consensus motifs in RHINO that are linked to phosphorylation by both PLK1 and the mitotic kinase CDK1 blocked the POL THETA/RHINO interaction.

In combination with the study by Gelot et al. (2023), the study by Brambati et al. (2023) provides a molecular mechanism for mitotic MMEJ, whereby PLK1 phosphorylation of RHINO and POL THETA promotes RHINO/PLK1 interaction, which directs POL THETA to mitotic DSBs. This interaction prevents micronuclei. Open questions for future investigation related to mitotic MMEJ include the following: to what extent does MMEJ complete during mitosis versus the ensuing G1 phase (Fig. 2, A and B), and the structural impact of RHINO binding and PLK1 phosphorylation on POL THETA mitotic function.

### Patch #2: Clustering pulverized chromosomes

If mechanisms that prevent micronuclei from forming in mitosis are unsuccessful, the cell must now face the potential for that structure to generate pulverized DNA, a catalyst for a genome-shattering event known as chromothripsis (Crasta et al., 2012). How pulverized DNA fragments from ruptured micronuclei can be neutralized has been examined in two recent studies (Lin et al., 2023; Trivedi et al., 2023). These studies have revealed a mechanism whereby the ruptured micronuclear DNA is clustered during interphase and then segregated together during mitosis into a single daughter nucleus, thus protecting the ruptured ends, at least temporarily, from seeding genomic instability.

Key players in the clustering process described by Lin et al. (2023) and Trivedi et al. (2023) are the following proteins: DNA topoisomerase II binding protein 1 (TOPBP1), cellular inhibitor of protein Phosphatase 2 A (CIP2A), and mediator of DNA damage checkpoint 1 (MDC1). TOPBP1 is a molecular scaffold that can localize to distinct mitotic structures including bridges between sister chromatids (Broderick et al., 2015). CIP2A, as its name implies, is best known for inhibiting the cell cycle regulatory Phosphatase PP2A and is activated through dimerization (Wang et al., 2017). Interestingly, PP2A can function as a negative regulator of DNA damage signaling (Chowdhury et al., 2005). MDC1 is a scaffolding protein that is rapidly recruited to sites of DNA damage.

Previous work showed that while MDC1 plays important interphase DDR roles, in mitosis it recruits TOPBP1 to chromosomal DSBs. Specifically, in human osteosarcoma cells subjected to chromosome breakage by X-ray irradiation, filamentous TOPBP1 structures were found to form at DSBs and to directly interact with MDC1 foci in mitosis. Cells expressing a mutant MDC1 that cannot bind to TOPBP1 exhibited sensitivity to DSBs, specifically in mitosis. These cells accumulated acentric DNA fragments that failed to properly segregate during mitosis, forming micronuclei. These results suggested that the MDC1–TOPBP1 axis stabilizes mitotic DSBs (Leimbacher et al., 2019). CIP2A was then identified as a TOPBP1-interacting protein that regulates TOPBP1 localization specifically in mitosis. CIP2A depletion in cells with mitotic DSBs led to acentric fragments and micronuclei, similar to prior findings regarding disruption of the MDC1–TOPBP1 interaction (De Marco Zompit et al., 2022). Of note, CIP2A was identified in a synthetic lethality screen in human cells for factors that are required in the absence of HR, consistent with a function for CIP2A outside of canonical interphase DDRs (Adam et al., 2021). These and other studies laid the groundwork for the idea that TOPBP1, CIP2A, and MDC1, independent of canonical DNA repair, could form a protein-based bridge between broken DNA ends during mitosis.

**Figure 3.**
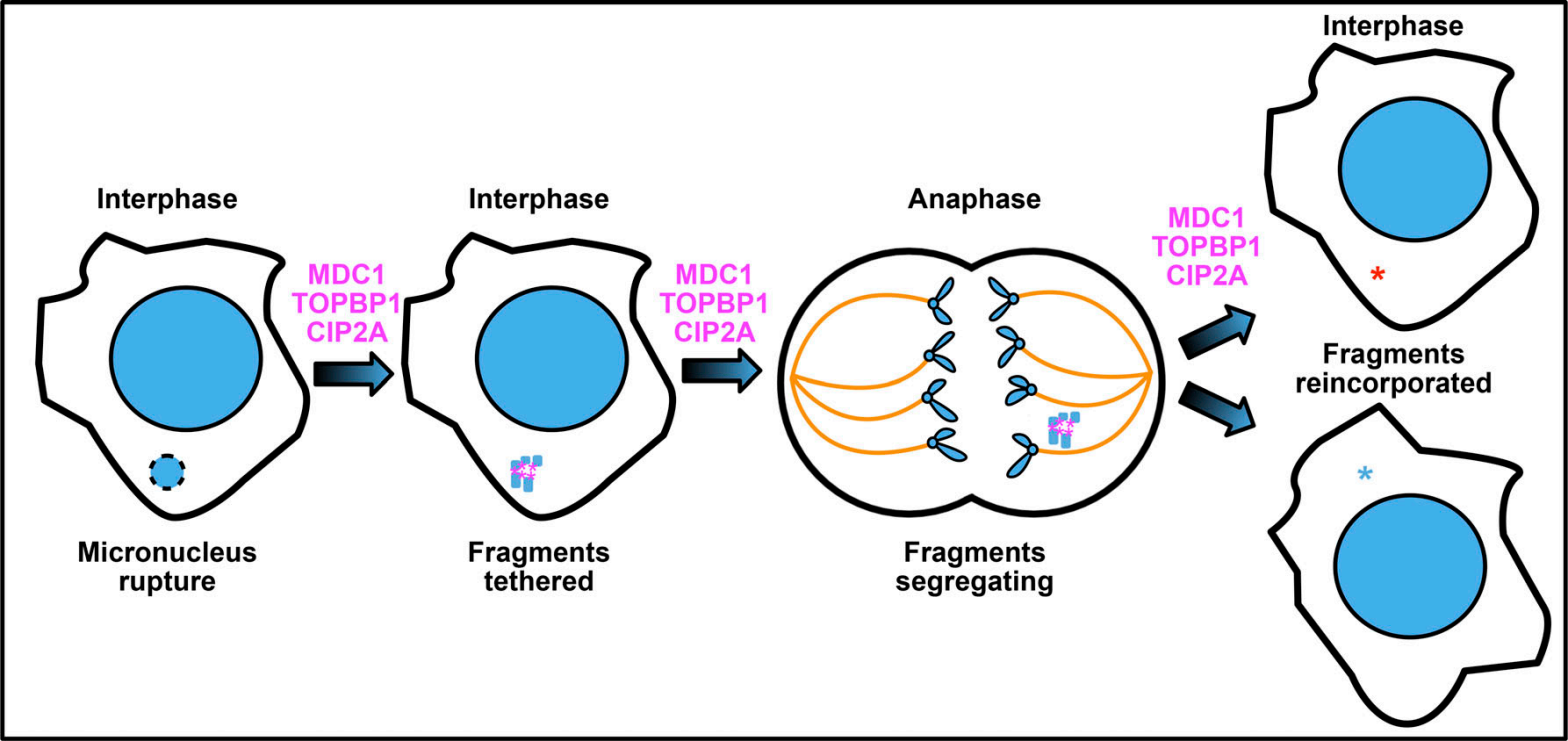
**Clustering of pulverized DNA ends from ruptured micronuclei and the ensuing mitosis.** Panels depict the rupture of a micronucleus in interphase and the subsequent mitosis. DNA, blue (large circle = nucleus, small circle with dashed outline = ruptured micronucleus and resulting fragments, smaller V-shaped structures = chromatids), spindle microtubules, yellow; tether, magenta. Blue and red asterisks track each presumptive and resulting daughter cell.

Both Lin et al. (2023) and Trivedi et al. (2023) examined MDC1, TOPBP1, and CIP2A in the context of chromosome fragments that arise upon micronuclear rupture (Fig. 3). To generate micronuclei, both groups employed multiple strategies, including a centromere inactivation strategy to induce acentric lagging Y-chromosomes in human colorectal carcinoma (DLD-1) cells. Using live imaging along with nuclease-dead Cas9-directed Y-chromosome fluorescent tagging, the authors tracked pulverized Y-chromosome fragments from micronuclei during the subsequent mitosis. This approach revealed that acentric micronuclear fragments remain tightly clustered together and segregate almost exclusively into a single daughter cell. Frequently, these clustered fragments are re-encapsulated in the nucleus, thus eliminating the micronucleus (Fig. 3). These findings present exciting evidence of an intrinsic ability of genomic fragments to cluster together upon micronuclear rupture, setting up the questions of how this micronuclear fragment clustering occurs and what the cellular consequences are of clustering versus non-clustering.

Localization studies by Lin et al. (2023) and Trivedi et al. (2023) then implicated CIP2A and TOPBP1 in clustering fragments from ruptured micronuclei. While CIP2A did not associate with interphase DSBs within the nucleus, Lin et al. (2023) identified both diffuse and punctate patterns of CIP2A that colocalized with TOPBP1 in multiple human cell lines within acentric fragments of ruptured interphase micronuclei. Interestingly, the groups somewhat differed regarding their findings on the role of γ-histone 2AX (γ-H2AX), a common marker of canonical interphase DNA DDR signaling. While Lin et al. (2023) report no impact on CIP2A-TOPBP1 recruitment to acentric fragments in ruptured micronuclei in H2AX deficient cells (Lin et al., 2023), Trivedi et al. (2023) found that TOPBP1 foci frequency was dramatically reduced in cells lacking γ-H2AX chromatin marks (Trivedi et al., 2023). The full recruitment and activation strategies concerning the CIP2A and TOPBP1 in ruptured micronuclei remain an area for future investigation.

Both Lin et al. (2023) and Trivedi et al. (2023) identified roles for CIP2A and TOPBP1 in acentric micronuclear fragment clustering. The groups subsequently investigated CIP2A and TOPBP1 function. Knockdown approaches by both groups included using degron alleles that can be inactivated at specific times, permitting careful study of the role of these proteins upon micronuclear rupture. Both CIP2A- and TOPBP1-compromised cells accumulated numerous distinct chromosomal fragments originating from ruptured micronuclei compared with control cells. Interestingly, whereas siRNA experiments targeting MDC1 performed by Lin et al. revealed only a minimal contribution of MDC1 to fragment clustering (Lin et al., 2023), Trivedi et al. found a role for MDC1 as well (Trivedi et al., 2023). MDC1 knockdowns performed by Trivedi et al. revealed a significant increase in the percentage of cells with dispersed micronuclear-derived acentric chromosome fragments after centromere inactivation as compared with controls (Trivedi et al., 2023). Moreover, Trivedi et al. found that TOPBP1-degron-induced clustering defects were rescued by wild-type TOPBP1 but not by mutants that lack MDC1 or CIP2A binding capability (Trivedi et al., 2023). Thus, while CIP2A and TOPBP1 were implicated in both studies, the role of MDC1 appears more complex and possibly context dependent.

Nevertheless, (Lin et al., 2023; Trivedi et al., 2023) both report a common mechanism of clustering of ruptured micronuclear fragments, and the downstream consequences of lacking this mechanism are in consensus between the studies. CIP2A depletion drastically reduced cell viability in micronucleated cells. This is an important result that may spur the development of therapeutic strategies that target the MDC1–CIP2A–TOPBP1 repair axis in cancer cells. It should be noted that while the patching mechanism reported by these studies clearly protects genomic integrity by protecting broken DNA ends and frequently erasing the micronucleus by re-encapsulation (Fig. 3), occasionally this mechanism fails and a micronucleus persists, which still poses a risk to the genomic integrity of the cell. Further, it is hard to imagine that the patched structure of numerous micronuclear DNA ends does not disrupt the function of the nucleus when it is re-encapsulated. Yet, a clear-cut impact on genome stability is evident from both reports. Metaphase spreads performed by Lin et al. (2023) suggest that CIP2A-dependent tethering is required to limit the loss of genetic material from pulverized chromosome fragments (Lin et al., 2023). Similarly, whole-genome sequencing performed by Trivedi et al. (2023) revealed an increase in both structural variant burden and copy number burden in CIP2A-deficient clones (Trivedi et al., 2023).

These studies reveal the importance of a newly appreciated MDC1–CIP2A–TOPBP1 mechanism that patches together acentric DNA ends from micronuclei and enables these ends to segregate together during the ensuing mitosis (Fig. 3), thus preventing genome catastrophe. Interesting future questions for study include the level of organization of the tethered DNA ends upon clustering, the precise role of the TOPBP1 filaments in stabilizing broken DNA ends, how the structure of patched micronuclear fragments facilitates segregation into daughter nuclei, and the long-term impact of reincorporating all those patched micronuclear DNA ends back into the nucleus.

### When to use which patch?

**Figure 4.**
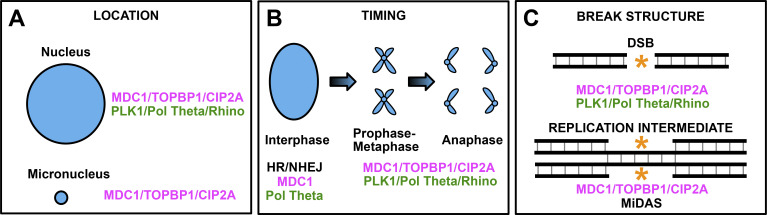
**Factors influencing distinct patch responses. (A)** Location of the DNA break may impact the molecular response. **(B)** Timing of the DNA break may impact the molecular response. **(C)** Structure of the DNA lesion may impact the molecular response. DNA, blue (large circle = nucleus, small circle = micronucleus, smaller V-shaped structures = chromatids), black ladder, double-stranded DNA, yellow asterisks mark regions of a DSB or DNA replication intermediates.

The two DSB patching mechanisms described here both impact mitosis and micronuclei (Figs. 2 and 3). The mechanisms appear to be activated sequentially. Gelot et al. (2023) and Brambati et al. (2023) describe a process that responds to DSBs that persist in the nucleus itself upon mitotic entry and prevent micronuclei from forming during that mitosis (Fig. 4 A) (Gelot et al., 2023; Brambati et al., 2023). But in the case that a micronucleus is successful in forming (Fig. 1), Lin et al. (2023) and Trivedi et al. (2023) describe a process that responds to DSBs in that micronucleus and prevents genomic catastrophe in the next mitosis (Lin et al., 2023; Trivedi et al., 2023).

### Molecular players

In addition to the differing events that trigger each patch response, another distinction is the molecular players (Fig. 4). Lin et al. (2023) and Trivedi et al. (2023) both depleted POL THETA and observed no impact on the clustering of pulverized micronuclear DNA (Lin et al., 2023; Trivedi et al., 2023). These findings suggest that the MDC1–TOPBP1–CIP2A patching response to ruptured micronuclei does not involve MMEJ. In addition, Brambati et al. (2023) observed additive effects on cell growth in HR-deficient cells codepleted of RHINO and CIP2A, which further suggests that MMEJ and MDC1–TOPBP1–CIP2A patching are separable processes (Brambati et al., 2023). If this is indeed the case, it raises interesting questions about Pol Theta. How is it that Pol Theta responds so efficiently to DSBs during mitosis, but is not required for a seemingly similar function in ruptured micronuclei? Pol Theta is capable of acting at DNA breaks in an extrachromosomal context, as it was recently shown to catalyze the formation of extrachromosomal circular DNAs in both *Drosophila* and human cells (Yang et al., 2023). Perhaps the extent of the damage (i.e., number of breaks) dictates the players involved. It seems likely that many chromosomal fragments from a ruptured micronucleus present a greater challenge to cellular patching machinery than a single acentric fragment. A ruptured micronucleus might be closer in scale to a full-on tire blowout, whereas tethering a single acentric fragment might be more analogous to repairing a nail hole in your tire.

Patching micronuclear fragments using MDC1–TOPBP1–CIP2A or initiating POL THETA-dependent MMEJ during mitosis may not be completely distinct processes. A common set of repair factors may exist upstream that preferentially recruits either CIP2A or POL THETA downstream depending on the damage. TOPBP1 appears a likely link. For example, in addition to its connections to CIP2A, TOBP1 has known RHINO binding capability (Cotta-Ramusino et al., 2011) and was correlated with RHINO in a dependency analysis performed by Brambati et al. (Brambati et al., 2023). Together, these results may suggest that TOPBP1 stabilizes RHINO to facilitate the recruitment of POL THETA required for MMEJ during mitosis (Cotta-Ramusino et al., 2011; Brambati et al., 2023). Similarly, Gelot et al. (2023) found that mitotic MMEJ by POL THETA requires not only PLK1 phosphorylation but also interaction with TOPBP1 (Gelot et al., 2023). Specifically, phosphorylated POL THETA is recruited to DSBs during mitosis by binding to TOPBP1 (Gelot et al., 2023). These results provide additional evidence that TOPBP1 is a common link between two different patching strategies. Further work that illuminates the full molecular networks in each process will reveal to what extent each event uses unique versus common machinery.

### Temporal considerations

The timing of the DNA break could be a major determinant of which response is activated (Fig. 4 B). As discussed in our overview of the response to micronuclear rupture (Patch #2), MDC1, TOPBP1, and CIP2A also partner within the nucleus to form bridging structures that stabilize broken chromosomes until the next G1 phase (Leimbacher et al., 2019; De Marco Zompit et al., 2022). This chromosomal MDC1–TOPBP1–CIP2A process closely mirrors the events that POL THETA may respond to. One proposed explanation for whether persistent nuclear DSBs are acted upon by chromosomal MDC1–TOPBP1–CIP2A is that MMEJ may respond to breaks that are carried over from interphase into the next M phase, whereas MDC1–TOPBP1–CIP2A may respond to breaks that arise in M-phase itself (Llorens-Agost et al., 2021a). Related to this model, cell type–specific gene expression and/or the status of canonical DDR mechanisms may also play a role. For example, MDC1 activity is influenced by both interphase DDR signaling and the mitotic activity of CIP2A and TOPBP1. Regarding the stage of mitosis when mitosis-related DDRs are activated, an interesting question for future study is the role of distinct phases of mitosis, as mitotic cells before or after the metaphase to anaphase transition are massively different in terms of the activity of key proteolytic events that drive mitotic progression. Along these lines, a recent study in *Drosophila* identified an early anaphase mechanism involving several components such as Polo, the ortholog of PLK1, whereby telomere–telomere connections between segregating sister chromatids are stabilized in neural stem cells with mitotic DNA breaks (Warecki et al., 2023).

### DNA break structure

One additional mechanism that likely distinguishes which mitotic DDR is engaged is the genomic location and nature of the DNA break (Fig. 4 C). Interphase DSBs that are carried over into mitosis often are the product of unreplicated DNA regions. Such unreplicated regions can be structurally distinct from other forms of DNA breakage. Mitotic DNA synthesis (MiDAS) is yet another mechanism that responds to such mitotic DNA damage (Bhowmick et al., 2023). MiDAS serves to complete DNA replication at unreplicated regions in mitosis. Future work will likely reveal structure-dependent mechanisms that respond to mitotic DNA damage.

### Even more ways to patch a flat

While the two responses highlighted here (Fig. 2 and Fig. 3) bring to light new mechanisms of mitosis-related DDRs, previous work has illuminated other such mitotic responses in diverse systems including insects, fungi, and cultured human cells (Carlson, 1938; Royou et al., 2010; Titen and Golic, 2008; Ishii et al., 2008; Kaye et al., 2004; Kanda et al., 2001). And to the extent to which molecular mechanisms have been identified in these other responses, some of the same players highlighted here are required. For example, *Drosophila* neuroblasts require DSB signaling to recruit Polo to mitotic DNA breaks (Royou et al., 2010; Derive et al., 2015; Landmann et al., 2020). There, Polo partners with the spindle assembly checkpoint proteins BubR1 to tether broken DNA ends of a mitotic chromosome and enable poleward segregation of the acentric fragment. This mechanism resembles the segregation pattern involving Pol Theta in *Drosophila* papillar cells; however, papillar cells do not require BubR1 to respond to mitotic DSBs (Bretscher and Fox, 2016). For further discussion of mitotic patches in diverse eukaryotes, we refer readers to a recent excellent review (Warecki and Sullivan, 2020).

In summary, the cell cycle stage of break induction, cell type, status of DNA break checkpoints, and the location and structure of DSBs all likely play an important role in how DNA damage is responded to in the context of mitosis.

## Conclusion

Compared with the study of interphase DDRs, much less is known about how mitotic cells with broken DNA can avoid genome catastrophe. The recent studies highlighted here, and many others, bring to light the important pathways that enable successful mitosis with broken DNA. A future challenge is to reveal the full constellation of molecular players and to identify the contexts in which each is called upon. Knowledge from this field can illuminate fundamental insight into, for example, how a single fertilized human zygote undergoes enough cell divisions to produce an adult with 40 trillion cells, how an injury can be rapidly repaired through faithful mitosis, and how cancers can be kept at bay. We are fortunate to have such good emergency roadside assistance.
